# Insight into the structure of decagonite – the extraterrestrial decagonal quasicrystal

**DOI:** 10.1107/S2052252520015444

**Published:** 2021-01-01

**Authors:** Ireneusz Buganski, Luca Bindi

**Affiliations:** a Faculty of Physics and Applied Computer Science, AGH University of Science and Technology, Krakow, Poland; b Graduate School of Engineering, Hokkaido University, Sapporo, Hokkaido 060-8628, Japan; c Dipartimento di Scienze della Terra, Università degli Studi di Firenze, Via La Pira 4, Firenze I-50121, Italy

**Keywords:** decagonal quasicrystals, X-ray diffraction, atomic structure refinement

## Abstract

A set of X-ray data collected on a fragment of decagonite, Al_71_Ni_24_Fe_5_, the only known natural decagonal quasicrystal found in a meteorite formed at the beginning of the Solar System, allowed us to determine the first structural model for a natural quasicrystal. It is a two-layer structure with decagonal columnar clusters arranged according to the pentagonal Penrose tiling. The structural model showed peculiarities and slight differences with respect to those obtained for other synthetic decagonal quasicrystals. Interestingly, decagonite is found to exhibit low linear phason strain and a high degree of perfection despite the fact it was formed under conditions very far from those used in the laboratory.

## Abstract 2

The crystal structure of decagonite, Al_71_Ni_24_Fe_5_, the only known natural decagonal quasicrystal found in a meteorite formed at the beginning of the Solar System is reported. The structural model obtained shows peculiarities and slight differences with respect to those obtained for other synthetic decagonal quasicrystals.

## 1. Introduction   

Quasicrystals (QCs) (Shechtman *et al.*, 1984 ▸; Levine & Steinhardt, 1984 ▸) short for quasiperiodic crystals, are solids violating the dogma of classical crystallography because their structure is ‘quasiperiodic’ rather than periodic; that is, their atomic distribution can be described by a sum of periodic functions with periods whose ratio is irrational. Their diffraction pattern consists of true diffraction peaks, the positions of which can be expressed as integer linear combinations of *n* integer linearly independent wavevectors where *n* is greater than the number of space dimensions. To date, QCs have been widely studied because of their potential industrial applications, such as hydrogen storage, hydride battery materials and coating of soft metals (*e.g.* Dubois, 2000 ▸).

Among the several synthetic quasicrystalline solids, the decagonal (d) Al_71_Ni_24_Fe_5_ phase (Tsai *et al.*, 1989 ▸) is particularly fascinating as it is the composition of decagonite, a natural-occurring quasicrystal (Bindi *et al.*, 2015*a* ▸, 2015*b* ▸). An extraterrestrial origin has been established for decagonite (Lin *et al.*, 2017 ▸), and its association with several high-pressure phases in the same meteorite suggests formation at high pressures and temperatures during an impact-induced shock (Meier *et al.*, 2018 ▸). Formation in asteroidal collisions in outer space for decagonite has also been recently corroborated by laboratory shock-experiments (Oppenheim *et al.* 2018 ▸). Here we report the structure determination of decagonite.

## 2. Decagonal quasicrystals and progress in the knowledge of their structure   

Decagonal quasicrystals (DQCs), apart from icosahedral quasicrystals (IQCs), are the most frequently observed intermetallic phases with a broken translational symmetry (Steurer, 2018 ▸). The decagonal point symmetry is either 10/*m* or 10/*mmm* (Rabson *et al.*, 1991 ▸). The tenfold direction is simultaneously perpendicular to the atomic planes. Therefore, the DQCs can be seen as a periodic stacking of the atomic layers with two-, four-, six- and eight-layer stacks within which atoms are quasiperiodically distributed (Steurer & Deloudi, 2014 ▸). The chemical bonding between atoms does not differ in-between or within the atomic layer (Steurer, 2006 ▸; Henley *et al.*, 2006 ▸). The typical unitary building units that atoms tend to locally form in the DQC like pentagonal bipyramids or icosahedral clusters, especially typical for six-layer periodic DQCs (Steurer *et al.*, 1994 ▸), are spanned across several atomic layers.

DQCs are categorized into two groups based on the elements constituting the structure: the Al–TM (TM: transition metal) and Zn–Mg–RE (RE: rare-earth element). The first DQC is metastable and belongs to the former group (Bendersky, 1985 ▸; Chattopadhyay *et al.*, 1985 ▸). It was found in 1985 in the rapidly cooled Al–Mn alloy. The first stable DQC was discovered in 1988 (He *et al.*, 1988 ▸).

The first structure analysis of a DQC by X-ray diffraction was performed on d-Al_65_Co_15_Cu_20_ by means of the 5D Patterson function. A total of 259 Bragg reflections were used to refine 11 parameters up to *R* = 9.8% (Steurer & Kuo, 1990 ▸), where the 5D space group was concluded to be *P*10_5_/*mmc* (reflection conditions: 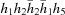 with *h*_5_ = 2*n* and 0000*h*_5_ with *h*_5_ = 2*n*, 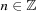). In general, two space groups satisfy the above mentioned extinction rules: centrosymmetric *P*10_5_ *mc* and non-centrosymmetric *P*10_5_ *mc*. Usually the centrosymmetric space group is assumed for the structure solution to impose the highest possible symmetry. The general assumption that all the known DQCs are centrosymmetric is not correct as the Co-rich d-Al_64_Co_22_Cu_14_ phase was determined to be non-centrosymmetric (Taniguchi & Abe, 2008 ▸).

### 2.1. Decagonal coordinate system   

The diffraction pattern of DQCs can be indexed with a set of four co-planar vectors directed towards diffraction peaks in the aperiodic plane and one additional vector directed along the periodic axis (*e.g.* Yamamoto, 1996 ▸ *a*). The components of four base vectors **d** ^*^_i_ of the reciprocal space are the following:
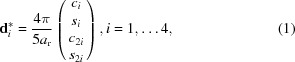
where 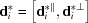. The term 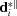 represents the coordinates of the reciprocal space base vector that form a 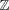-module of rank 4 in physical space where the diffraction pattern is observed. The 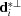 components simply extend the vector space and are defined within perpendicular space. The abbreviated notation in equation (1) is 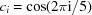 and 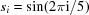. The term *a*_r_ defines the edge-length of the rhombus in the rhombic Pentose tiling (RPT) (Penrose, 1974 ▸). The vectors **d**_*i*_ of the direct space can be found by the known relation 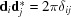.

The definition of the projection matrix **W** in direct space that allows us to project the higher-dimensional (*n*D) model of the structure into 2D representation is:
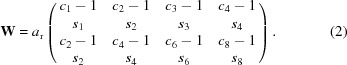
The relation between symmetry-adapted vectors **d**_*D*_ of 4D space and the Cartesian coordinates **d**_*V*_ that decompose the real-space and perpendicular-space coordinates is 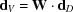, where 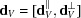. Vectors **d**_*D*_ are such that when pointing towards the vertices of the 4D unit cell, their components are either 1 or 0.

We do not go into detail on the *n*D representation and how to use the projection method. Details about the projection and section methods for QCs can be found elsewhere (Katz & Duneau, 1986 ▸; Kalugin *et al.*, 1985 ▸; Elser, 1986 ▸; Bak, 1985 ▸).

### 2.2. Structure modeling techniques   

Nowadays, there are three dominant methods of constructing the atomic model of the quasiperiodic structure for the refinement. Two are closely related: the atomic surface-modeling technique used, for example, for d-Al_70.6_Co_6.7_Ni_22.7_ by Cervellino *et al.* (2002 ▸) and the cluster-embedding method applied, for example, by Takakura *et al.* (2001 ▸) for d-Al_72_Co_8_Ni_20_. Both methods utilizes the *n* D apparatus. The third method is based on physical space where no atomic surface is considered during the refinement (Wolny, 1998 ▸; Wolny *et al.*, 2014 ▸). However, it is possible to lift the structure to higher-dimensional space and express the structure as a concept of *n*D space.

For the purpose of this research, the physical space structure refinement was exploited. This approach was utilized recently for several DQCs and also IQCs, resulting in the first detailed model of the Bergman-type IQC (Buganski *et al.*, 2020*b* ▸). Even so, the approach is questioned as to why the local ordering of atoms is prioritized more than long-range order, the presumption is further confirmed by the high-resolution electron microscopy at least for DQCs (Li *et al.*, 2016 ▸; Hiraga, 2002 ▸). The local clustering of atoms is also widely accepted for IQCs (Takakura *et al.*, 2007 ▸).

## 3. Experimental   

### 3.1. Sample characterization techniques   

The natural DQCs studied here come from Grain 126 of the Khatyrka meteorite (Lin *et al.*, 2017 ▸; MacPherson *et al.*, 2013 ▸). It was investigated by means of transmission electron microscopy (TEM), single-crystal X-ray diffraction, scanning electron microscopy energy dispersive spectrometry (SEM-EDS) and electron microprobe wavelength dispersive spectrometry (EMP-WDS) techniques.

### 3.2. Transmission electron microscopy   

A small amount of decagonite powder from Grain 126 was placed on a Cu mesh TEM grid (300 mesh, 3 mm in diameter) that was previously covered by a thin carbon layer (support film). EDS data were obtained using Evex NanoAnalysis System IV attached to the Philips CM200-FEG TEM. A small electron probe of 20–100 nm was used with a count rate of 100–300 cps using an average collection time of 180 s. Quantitative analyses were taken at 200 kV and are based on the use of pure elements and the NIST 2063a standard sample as a reference under identical TEM operating conditions. The average of the three-point analysis gave, on the basis of 100 atoms, the formula Al_74(3)_Ni_20(2)_Fe_6(2)_.

### 3.3. Single-crystal X-ray diffraction   

A single fragment of decagonite (7 × 8 × 14 µm) was selected to perform X-ray diffraction studies. Such studies were carried out with both an Oxford Diffraction Xcalibur 3 CCD single-crystal diffractometer, operating with Mo*K*α radiation (λ = 0.71073 Å) and with 100 s per frame exposure time, and an Oxford Diffraction Excalibur PX Ultra diffractometer equipped with a 165 mm diagonal Onyx CCD detector at 2.5:1 demagnification operating with Cu*K*α radiation (λ = 1.5406 Å) and 60 s per frame. The data collected with Mo*K*α radiation are presented here. We have determined the lattice parameters to be 2.450(8) Å (the edge-length of the rhombus) and 4.105(7) Å (along the periodic direction). In total, 737 independent diffraction peaks were collected with a spherical absorption correction and *R*_int_ = 0.096.

**Figure 1:**
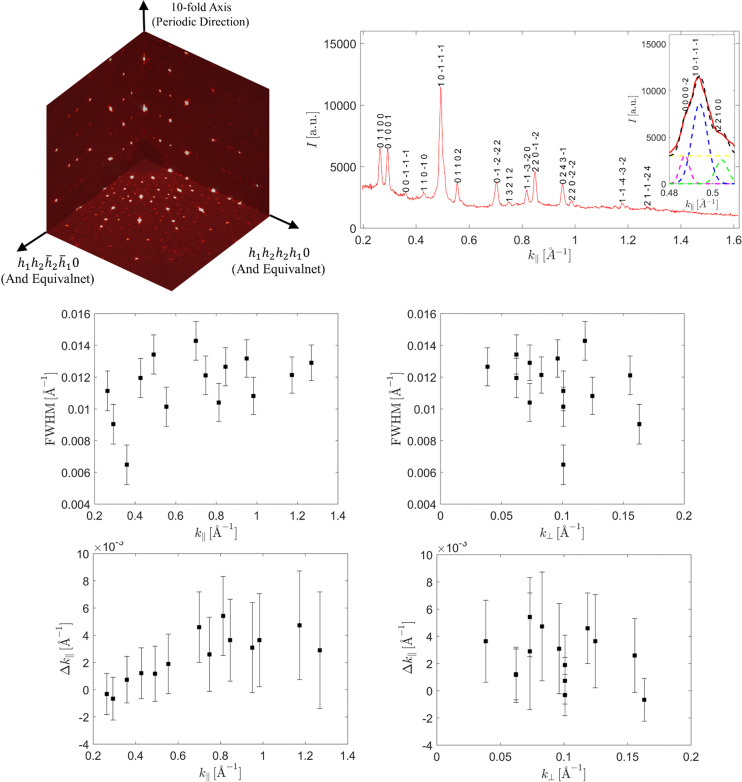
Analysis of the diffraction data. (Top) The planar sections through the 3D diffraction pattern and its representation in the 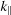 domain are presented. The superstructure peak is circled with a yellow-dotted line. The profile of the peak 10111 [indexed in setting (1)] indicates it is in fact assembled out of three peaks that are not separated due to the high width of the diffraction peaks. (Mid and bottom) FWHM and the peak shift with respect to 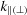. Large experimental uncertainty of the measured peak characteristics makes quantitative analysis of the phason and phonon strain impossible.

In Fig. 1 ▸ the summary of the sample characterization based on single-crystal diffraction is presented. Three perpendicular planar sections through 3D Fourier space are shown. No substantial diffusive scattering is detected in either plane, a frequent occurence for a synthetic d-Al–Cu–Co indicating the existence of superstructure (Kuczera *et al.*, 2012 ▸) or antiphase domains in the aperiodic plane of the structure (Bogdanowicz, 2003 ▸). In the plane [*h*_1_ *h*_2_ *h*_2_ *h*_1_0] × [00001] sharp spots in between periodic series of peaks can be sporadically found (one of these peaks is marked with a yellow circle).

The analysis of the strains in the sample of decagonite was carried out after the 3D Fourier space was transformed into a powder diffraction diagram. The diagram was indexed with vector setting (1). The peaks are broad with poor peak separation as is evident from peak 101 1 1. It is composed of three large peaks: 101 1 1, 00002 and 22100 which were fitted to the peak profile with Gaussian curves. After indexing, the physical and perpendicular space analysis of the strains in the crystal was performed. We were interested to see if the sample exhibited quenched linear phason strain.

For random and isotropic distribution of phason and phonon strains the FWHM of the Bragg peak depends on both the physical space (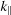) and the perpendicular space (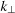) components of the wavevector (Lubensky *et al.*, 1986 ▸; Boudard *et al.*, 1996 ▸; Yamamoto *et al.*, 2004 ▸). Both effects can be deconvoluted for peaks with large 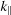 and small 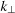; the peak width is mostly affected by the phonon strain. In the reverse situation, the FWHM is mostly impacted by the phason strain. The FWHM, which is a result of the convolution of the phonon and phason strain distribution, both in the form of Gaussian functions, is 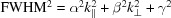, where α, β and γ are parameters. The fit with a least-square method yields α = 0.0031(32), β = 0.006(13) and γ = 0.1233 (86). Only γ (experimental momentum resolution) was determined with an uncertainty lower than the parameter value. Therefore, based on the diffraction data, we cannot determine quantitatively the parameters of the phason and phonon strain.

Based on the peak shift the shear deformation (linear phason strain) of the hypercrystal can be estimated (Goldman & Kelton, 1993 ▸; Gratias *et al.*, 1995 ▸). However, in our case the data quality is not sufficient to determine the phason strain matrix. For most detected peaks the peak shift is not different from zero shift by more than one standard deviation. In conclusion, the diffraction data do not allow us to make claims about the existence or absence of linear phason strain.

### 3.4. Scanning electron microscopy   

The same fragment studied by single-crystal X-ray diffraction was then analyzed by means of an FEI *QUANTA* 200 FEG environmental-ecanning electron microscope equipped with an Oxford INCA Synergy 450 energy-dispersive X-ray microanalysis system, operated at 15 and 5 kV accelerating voltage, 140 pA probe current, 2000 cps as average count rate on the whole spectrum, and a counting time of 60 s; and with a Zeiss EVO MA15 scanning electron microscope coupled with an Oxford INCA250 energy-dispersive spectrometer, operating at 20 and 5 kV accelerating voltage, 500–150 pA probe current, 2500 cps as average count rate on the whole spectrum, and a counting time of 500 s.

### 3.5. Electron microprobe   

After the semi-quantitative SEM analyses, the same fragment used for the X-ray study was studied with a Jeol JXA-8200 electron microprobe operating at an accelerating voltage of 15 kV, beam current of 20 nA and a beam diameter of 1 µm. Variable counting times were used: 30 s for Al, Ni and Fe, and 60 s for the minor elements Mg, Si, Cr, P, Co, Cu, Cl, Ca, Zn and S. Replicate analyses of synthetic Al_53_Ni_42_Fe_5_ were used to check accuracy and precision. The crystal fragment was found to be homogeneous (five-point analyses on different spots) within analytical error. The standards used were: metal-Al (Al), synthetic Ni_3_P (Ni, P), synthetic FeS (Fe), metal-Mg (Mg), metal-Si (Si), metal-Cr (Cr), metal-Co (Co), metal-Cu (Cu), synthetic CaCl_2_ (Ca, Cl) and synthetic ZnS (Zn, S). Mg, Si, Cr, P, Co, Cu, Cl, Ca, Zn and S were checked and found to be equal to or below the limit of detection (0.05 wt%). On the basis of 100 atoms, the formula can be written as Al_70.2(3)_Ni_24.5(4)_Fe_5.3(2)_.

## 4. *Ab initio* structure solution of decagonite   

The first models of DQCs: d-Al–Co–Cu and d-Al–Ni–Co, were obtained by the 5D Patterson method and direct HRTEM imaging (Steurer & Kuo, 1990 ▸; Burkov, 1991 ▸). Nowadays, the initial structure can be successfully solved by the low-density elimination method (LDEM) (Shiono & Woolfson, 1992 ▸) or the charge flipping algorithm (CF) (Oszlányi & Sütő, 2004 ▸). The former algorithm is implemented in both the *QUASI07-08* package (Yamamoto, 2008 ▸) and the *SUPERFLIP* software (Palatinus & Chapuis, 2007 ▸), whereas only the latter is implemented in *SUPERFLIP*.

The dataset of 737 symmetrically independent diffraction peaks collected on decagonite was used for the phase retrieval in *SUPERFLIP*. Constraints in the form of symmetry operations available in the space group *P*10_5_ *mmc* were used. The group generators: the tenfold screw axis {*C*_10_∣**d**_5_}, a glide plane {σ∣**d**_5_} and the inversion {*I*∣0} make in total the order of the group equal to 40. The *ab initio* structure solution with *SUPERFLIP* resulted with a crystallographic *R* = 14.9%.

### 4.1. The *n*D analysis   

**Figure 2:**
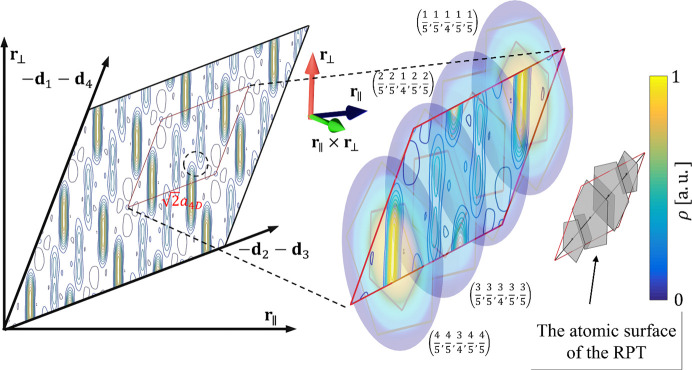
2D section through the 4D electron density map calculated on the basis of the 737 phased diffraction peaks. Two atomic layers of the physical space are superposed here. Four atomic surfaces centered along the [1111] direction are identified. They divide the long-body diagonal of the 4D unit cell into five equal sections. In the magnified picture of the section through one 4D unit cell, the distribution of the electron density within four atomic surfaces is emphasized. The smaller picture with four pentagons of the RPT is given as a guide. The maximal electron density is located in the first and fourth pentagons suggesting the distribution of TM atoms. The parameter *a*_4D_ is the edge-length of the 4D unit cell, which in the case of decagonite is equal to 5.478 Å.

In Fig. 2 ▸ a 2D section through the 4D electron density is shown. The section was calculated to contain the long-body diagonal direction [1111] and contains both atomic layers of the structure. Two vectors of the 4D vector base are spanning the section: the vector −**d**_1_ − **d**_4_ and the vector −**d**_2_ − **d**_3_ [the definition of vectors according to equation (1)]. The outline of the 4D unit cell was plotted with a red line. Four atomic surfaces lie equidistant on the long-body diagonal direction. At this time it is possible to exclude the model of d-Zn–Mg–Dy as an isostructure. It requires the mid-edge atomic surface that is not visible in the given section (Ors *et al.*, 2014 ▸). The structure model must be founded on the idea that only two atomic surfaces are generated (the two remaining are symmetrically dependent owing to the inversion symmetry). Additionally, we can see that the higher electron density is localized in the atomic surface placed in the position (1/5, 1/5, 1/5, 1/5) (excluding the coordinate along the periodic direction – the mid-coordinate in Fig. 2 ▸). This means most of the TM atoms are gathered in this atomic surface, occupying the center and separated from Al atoms occupying the outer part. The Al atoms, which possess a lower X-ray scattering power, occupy the other, (2/5, 2/5, 2/5, 2/5) localized, atomic surface. Such a dominant agglomeration of one kind of atomic species is known for all DQCs. All these features are satisfied by Yamamoto’s model of the d-Al–Cu–Co (Yamamoto, 1996 ▸ *b*); however, in that model 20 Å decagonal, fully symmetrical clusters are prioritized. It will be further proven those assumptions are invalid for the structure of decagonite. To further confirm the distribution of the electron density on the atomic surfaces, we have calculated the electron density in plane of the atomic surfaces. The shapes created by the electron density agree well with the archetype atomic surface of the RPT. In the calculated electron density, we made an outline of the idealized pentagons as a guide for an eye. TM atoms are located within the small pentagon, whereas the Al atoms occupy the area contained within the shape of the τ = (1 + 5^1/2^)/2 larger pentagon. Previous studies confirm the correctness of such distribution (Yamamoto *et al.*, 1990 ▸; Steurer *et al.*, 1993 ▸). The RPT can be a good quasilattice for a construction of the structure model, especially as it has already been successfully employed for the structure solution of d-Al–Cu–Me (Me = Co, Ir, Rh) (Kuczera *et al.*, 2012 ▸) and d-Al–Ni–Co superstructure type I (Kuczera *et al.*, 2011 ▸).

Based on the section through the 4D unit cell, it is possible to derive conclusions on the phasonic disorder. It is known that the physical-space atomic coordinates are created by the intersection of the physical space with the atomic surface. Along the physical space direction 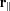 a short interatomic distance is created, when intersecting the atomic surfaces in the area marked with a black-dotted circle. These two positions cannot be occupied simultaneously, therefore an atom is able to move freely in this area. It is a phason flip site.

### 4.2. The real-space analysis   

**Figure 3:**
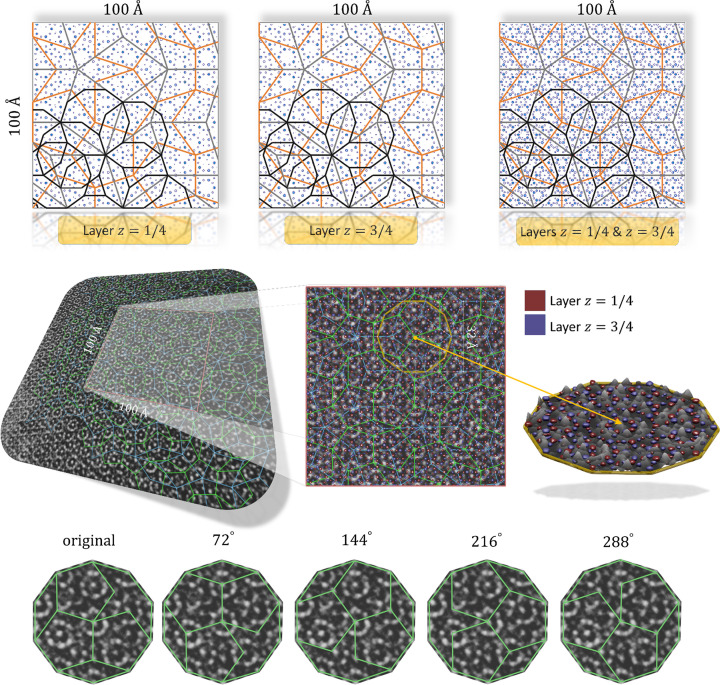
(Top) 2D section through the physical space electron density map perpendicular to the tenfold direction. The RPT (gray) and PPT (orange) are plotted over the contour plot. Hiraga clusters (black) are centered at the vertices of the PPT. (Bottom) HRTEM image of the decagonite structure with both RPT and PPT plotted over the image for guidance. Plotted tiling are τ times deflated in comparison with tilings in top contour plots. The isosurfaces of the electron density are compared with the magnified patch of the HRTEM image further supporting the agreement between the obtained *ab initio* structure solution and the real-space structure. The positions of clusters that are not centered in the PPT are located in the positions of the rearranged tiling: these are phason-flip-equivalent positions.

Much more can be derived about the 3D structure of the DQC on the basis of the physical-space sections through the electron density. First of all, the structure shows the length of the period along the tenfold axis equal to *A*_per_ = 4.105 Å, therefore the structure is a two-layer periodic structure. The layers at *z* = 1/4 and *z* = 3/4 and their combined projections along the tenfold direction are presented in Fig. 3 ▸. The notation *z* = 1/4 means that the layer is located at the level 1.4*A*_per_ ≃ 1.03 Å. The analogous is true for *z* = 3/4. The orange line marks the PPT (pentagonal Penrose tiling) with an edge-length of 19.73 Å and the gray line marks the RPT with an edge-length of 27.17 Å.

One of the basic units distinguished for DQCs based on the HRTEM images is the Hiraga cluster (Hiraga *et al.*, 1991 ▸), marked in Fig. 3 ▸ with a black line. The center of each decagon is placed at the vertices of the PPT with an edge-length of 19.73 Å. It is a decagon with a diameter of 31.93 Å originally discovered for d-Al–Cu–Co based on HRTEM images. There are three ways the cluster overlaps: they share one common edge, overlay making a hexagonal shape or overlay creating an irregular octagon, when Hiraga clusters are placed at the vertices of a rhombus in PPT along the short-body diagonal. The distances between clusters are 31.93 (19.73 Å × τ), 19.73 and 12.19 Å (10.73 Å/τ), respectively, where τ is the golden mean ratio.

The atomic decoration for rhombi depends on the atomic layer. Corresponding rhombi that stack one over another have two different atomic decorations. However, certain rhombi with the same orientation from layer *z* = 1/4 have the same decoration as the rhombi from layer *z* = 3/4 but are additionally inversed with respect to the geometrical center of the rhombus. After two layers of atoms are projected along tenfold axis, the single atomic decoration can be seen. It is the aftermath of the symmetry of the structure that possesses the screw axis along the tenfold direction.

Further confirmation that both PTs are correct quasilattices for the description of the atomic structure of decagonite comes from the investigation of the HRTEM images. Both ideal PPT (green) and RPT (blue) are plotted over the image to prove that visible decagonal clusters are ideally located in specific positions within both tilings. It is best visible for the PPT as the centers of the decagons follow the vertices of the PPT. Not all the clusters are centered in the vertices of the plotted PPT. Remaining clusters are centered in the vertices of the locally rearranged PPT. In the bottom part of Fig. 3 ▸, five orientations of the decagonal motif from PPT are presented. All the visible clusters are centered in the vertices of the PPT, but to do so, the pattern must be consecutively rotated by 72°. It is a phason flip. It must be stated that such a behavior is true for all the quasicrystals, even those with no linear phason strain. The ambiguity of plotting the PPT is also visible in the isosurface plot. In this case the edge length of the PPT is 12.19 Å and the edge length of the RPT is 16.79 Å which correspond to both tiling being τ times deflated with respect to tilings plotted over the electron density sections. The resolution of the HRTEM images better visualizes decagonal clusters with a diameter of 12.19 Å that is the diameter of τ^2^ times smaller cluster than Hiraga cluster. Additionally, the isosurface map was overlaid with the HRTEM image confirming the match. These are two independent techniques that show exactly the same result, confirming that long-range atomic order follows the PT. The exemplary Hiraga cluster was cut from the image and magnified to emphasize that isosurfaces ideally localize in the spaces of the HRTEM image corresponding to atomic species.

**Figure 4:**
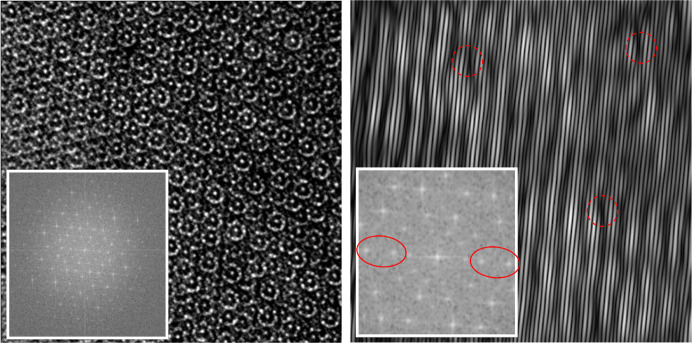
600 × 600 px HRTEM image and its Fourier transform (inset in left image) presented in log scale. The inverse Fourier transform for the co-linear peaks of the reciprocal space, encircled with a red continuous line in the inset (right) magnified Fourier transform, was calculated in order to show the Ammann lines. (Right) Image shows wavy lines indicating the phonon strain. A few instances of dark patches are marked with red dotted circles. The line pattern is continuous on both sides of the black area hence there is no jag that can be ascribed to linear phason strain.

Even so the agreement between those two techniques is excellent, small deviations from ideal tiling can be detected in the HRTEM image. The differences are caused by a conventional phonon strain. The clusters in the HRTEM image are displaced from perfect tiling positions (up to couple of Ångstroms): a clear indication of phonon strain (Socolar, 1986 ▸). More detailed analysis based on direct imaging is shown in Fig. 4 ▸. The effect of both phonon and phason strain is best visible in QCs when analyzing the Ammann lines. The pattern of Ammann lines was calculated following the analysis provided in the work by Freedman *et al.* (2007 ▸) and Lifshitz (2011 ▸). Four peaks of the diffraction pattern of the HRTEM image were filtered within a radius of 5 px and inverse Fourier transformed resulting in a pattern of lines. The lines are wavy, indicating the existence of the spatially varying phonon strain. Too an extent, the waviness can be ascribed to the image contrast modulation but the phonon strain is also confirmed by the displacement of cluster centers from the perfect tiling positions. There are black areas that could be interpreted as dislocations (exemplary areas are marked with red dotted circles in Fig. 4 ▸) but after closer examination the broken lines continue through the black spot. If linear phason strain or dislocation were observed, the line would be jagged, without continuation on the other side. For selected diffraction peaks we could not point unambiguously the position of the mismatch that could be ascribed to the linear phason strain. The darker patches are probably the result of the varying intensity of the HRTEM image. By selecting different pairs of τ-related diffraction peaks all seem to be perfectly in-line within the image resolution, meaning there is no visible linear phason strain.

### 4.3. The initial model   

**Figure 5:**
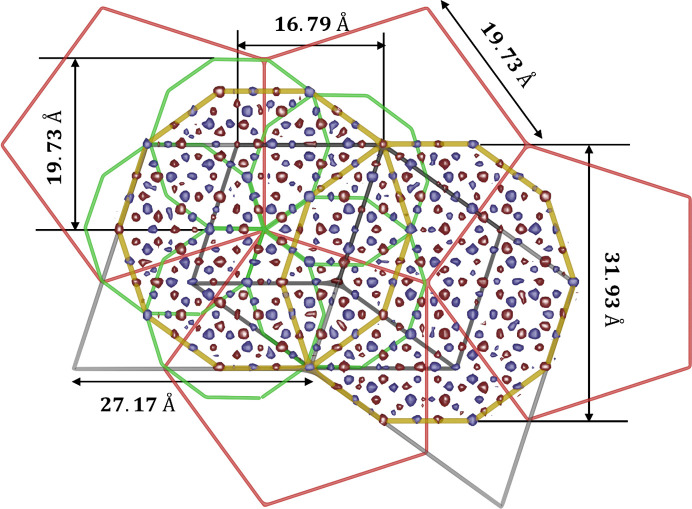
Relations between different tilings and clusters found in decagonite. The atomic decoration of the basic building blocks of the structure, the thick and thin rhombi of the 16.79 Å RPT, are found based on the relation to the Hiraga cluster.

The atomic decoration of two rhombi of the RPT can be found by considering the relations between the tilings derived from mutual local derivable class (Baake *et al.*, 1991 ▸) and the Hiraga cluster. The relations are presented in Fig. 5 ▸. Hiraga clusters are centered at the vertices of the PPT and their overlap forms a co-shared irregular hexagon. The Hiraga cluster is embedded (apart from the half hexagonal motif which can be restored by a mirror applying a mirror symmetry) within one thick rhombus with an edge-length of 17 Å. If the atomic model was based on a rhombus of this size, the number of parameters would be almost the same as the number of available diffraction peaks. It is possible to choose the τ-deflated tiling as the atomic decoration of a larger rhombus is constrained by the atomic decoration of downscaled rhombi and the basic asymmetric part of the Hiraga cluster is still contained within a thick rhombus. The smaller tiling yields a reasonable number of parameters for the refinement. The same approach has also been used for Al–Cu–*M* (*M* = Rh, Ir, Co) d-phases (Kuczera *et al.*, 2012 ▸). In Fig. 5 ▸ the so-called Deloudi clusters (Deloudi *et al.*, 2011 ▸), with a diameter of ∼20 Å proposed for d-Al–Cu–Co, are plotted in green. It can be seen that the Hiraga cluster is a supercluster of five overlapping Deloudi clusters.

The chosen space group for decagonite does not imply the tenfold symmetry of the Hiraga cluster in the final model. The highest possible symmetry expected for decagonal clusters after the refinement is the *m* symmetry as the diagonal (long for a thick rhombus and short for a thin rhombus) of the rhombi of the RPT has a mirror symmetry. The broken tenfold symmetry of the decagonal cluster was previously discussed by many scientists and reported for real-life systems (Saitoh *et al.*, 1997 ▸, 1998 ▸) including d-Al–Co–Ni. Some scientists even prefer to consider the symmetry to be locally different (Yan & Pennycook, 2000 ▸).

**Figure 6:**
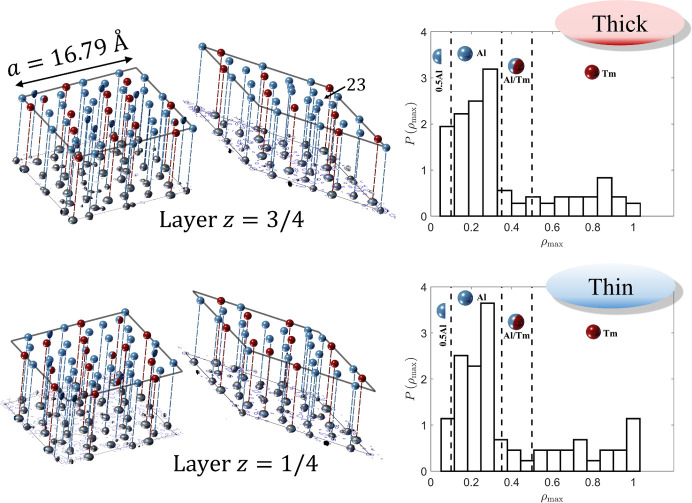
Initial atomic decoration of two rhombi of the RPT at each atomic layer. Color coding is as follows: red – TM, blue – Al, blue/red – mixed atom, blue half-sphere – partially occupied site by Al. Atom 23 (see the supporting information for details) is indicated. The atom potentially causes the point density of the model to be too high. The distributions of electron density (ρ_max_) in the positions of local maxima are plotted for thick (top) and thin (bottom) rhombi. The electron density is normalized by the maximal electron density within each rhombus. Two emerging sectors correspond to two atomic species likely to be present in the structure.

The initial atomic decoration was found by assigning atomic species to maxima of the electron density map in a region corresponding to thick and thin rhombi of the RPT with an edge-length of 16.79 Å. Such an electron density map enclosed within two rhombi is presented in Fig. 6 ▸. The threshold for the electron density maximum was tuned to reject short interatomic distances. Among two maxima that were too close to each other, the stronger one was always accepted. We found that the reasonable threshold was at the level of 1/30 of the electron density maximum. In total, 57 atoms were listed of the asymmetric part of the thick rhombus and 37 atoms of the thin rhombus. The asymmetric part is always half the volume of each rhombi cut along the diagonal.

The remaining issue is the distribution of chemical species. Due to the fact that Ni and Fe are indistinguishable by X-ray diffraction, the structure was solved as a pseudo-binary Al-TM system. The initial atomic decoration could be achieved by plotting the distribution of the electron density maxima. The distributions obtained are plotted in Fig. 6 ▸ where the electron density is normalized to the electron density maximum. It is evident that there are two distributions for each rhombus corresponding to two differentiable atomic species: Al and TM. Three thresholds are considered. For the maxima above 0.5ρ_max_ TM is assigned. Between 0.35ρ_max_ and 0.5ρ_max_ the mix site with 50/50 fraction of elements is assigned. The exact occupancy is further refined. Below 0.35ρ_max_ all the positions are assumed to be Al. Additionally, if the electron density in a selected position is below 0.1ρ_max_, a partial occupancy is assigned. The initial composition of the atomic structure is close to that obtained by electron microprobe and is equal to Al_71_TM_29_. The crystallographic *R* factor of the starting model is equal to 39%. That value, though large for inorganic crystals, does not imply that the model is flawed. Firstly, at this stage of the investigation the model was not refined. Secondly, the phason disorder (phason flips) is known to be a dominant factor affecting the intensity of the diffraction peak (Buganski *et al.*, 2019 ▸).

It is worth pointing out that all the atoms lay perfectly on two atomic layers *z* = 1/4 and *z* = 3/4 without any implication of possible puckering. This is important because the *z* coordinate is predetermined and it is redundant to refine it further. Previously known d-phases all appear to manifest stronger or weaker displacement of atoms from the atomic layers being at the same time mirror planes in the *P*10_5_/*mmc* symmetry.

## 5. The structure refinement   

The structure refinement is based on real-space modeling. The real-space structure refinement based on the average unit cell approach has been used before for decagonal structures (Kuczera *et al.*, 2012 ▸, 2011 ▸). Its main principle is the construction of the atomic distribution function (Wolny, 1998 ▸; Wolny *et al.*, 2018 ▸; Buczek & Wolny, 2006 ▸).

During the structure refinement, 256 free parameters were refined, including atomic coordinates, the phononic atomic displacement parameter (ADP), the phasonic ADP in a general Debye–Waller formula (Bancel, 1989 ▸; Lubensky *et al.*, 1986 ▸) (one parameter for a whole structure), one extinction parameter (Coppens & Hamilton, 1970 ▸) and a scale factor between the experimental and calculated structure amplitudes. Only isotropic ADPs are considered. Additionally, for the mixed atoms the partial occupancy probability for each element was refined, with the restriction that the sum of all values has to equal 1. The positions of atoms occupying the vertices of the rhombi are not optimized. Edge-bound atoms are allowed to move along the edges only. Since we refine the pseudo-binary Al-TM system, the weighted atomic form factor for TM of Ni:Fe = 24:5 has been used.

The parameters are refined against 737 symmetrically independent diffraction peaks satisfying the condition ∣*F*∣ > σ(∣*F*∣) that makes the reflection-to-parameter ratio ∼2.88. The given ratio, although low for an inorganic structure, allows us to perform the structure refinement. The problem of the low reflection-to-parameter ratio is known in the crystallography of QCs. The number of free parameters can be artificially managed in the *n*D approach with the arbitrary coarse subdivision of the atomic surface. This however imposes unrealistic constraints on atoms that have no confirmation in reality.

The refinement of the structure was conducted with the use of in-house code written in the *Matlab* software environment. The library *fmincon* was used to optimize the parameter of the structure with the interior-point algorithm as a solver. In the case of decagonite, the optimization function was the crystallographic *R* factor. The other evaluation function that was calculated, but not used for the refinement itself, was the weighted *wR* facor with 1/σ(*F*)^2^ weighting scheme.

The optimization strategy for the refinement is required. Due to the complexity of the structure, several refinement cycles with a subset of all free parameters must be completed before running a full set of free parameters. In the first run, only atomic coordinates are optimized. The locations of maxima that served to define the atomic decoration of rhombi are determined up to 0.05 Å – the grid resolution. The initial atomic positions are therefore exposed to high initial fluctuations that also affect the initial value of the *R* factor. After the first cycle *R* ≃ 23% was obtained, a value indicating that the model was potentially correct. In the next cycle, the phononic ADPs are refined. After crude estimation of parameters is achieved, the extinction parameter is released. Then the refinement cycles are cyclically repeated until finally all the parameters are released. The phasonic ADP parameter of the general Debye–Waller factor is optimized at every step of the refinement. It is a global parameter that strongly affects the calculated values of diffraction amplitudes. The number of diffraction peaks and their range of magnitudes do not allow us to derive more detailed information of the intrinsic phasonic disorder (Buganski *et al.*, 2020*a* ▸) except a crude evaluation in the picture of the Gaussian function.

**Figure 7:**
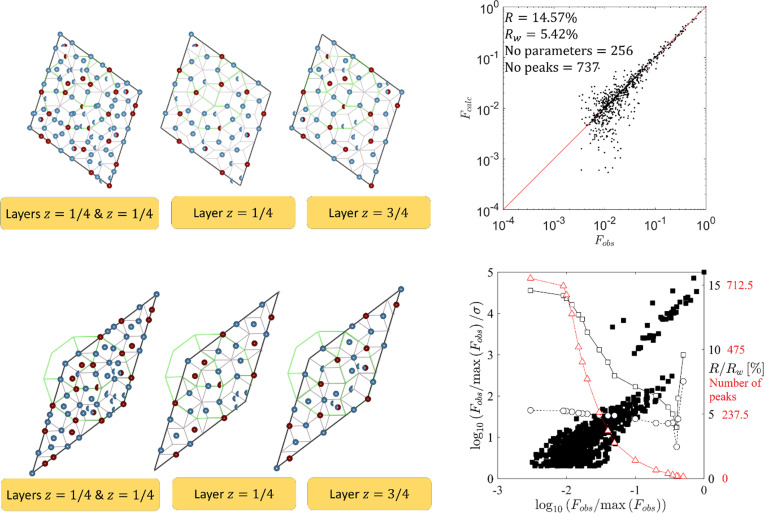
Refined atomic decoration for each type of rhombi in the RPT and every atomic layer. The color coding is the same as in Fig. 6 ▸ with an addition of a red half-sphere defining the partially occupied TM site. The basic 2.45 Å RPT is plotted within every rhombus confirming the positions of atoms follow the specific sites of rhombi. The Gummelt cluster is plotted for guidance. The correlation plot *F*_calc_ versus *F*_obs_ shows good agreement of the model with experimental data. The distribution of diffraction peak errors shows a two-domain behavior. The value of the *R* factor (standard and weighted) decreases with the decreasing number of weak reflections taken for calculations.

After the structure refinement, which resulted in *R* = 14.57%, no short atomic distances were observed, with the exception of the partially occupied atomic sites that are localized in the high-symmetry positions of the rhombi (Fig. 7 ▸). We have allowed for only a small shift of an atom from the original position (<1 Å in each cycle of the refinement program) that restrains the atoms from freely sliding within the structure. We can observe a few instances of Al/TM mixed occupancies with a dominant fraction of Al, in one case reaching over 98%. The mixed sites are located mostly along the mirror plane that is a collateral of the optimization procedure. The final composition after the refinement was concluded to be Al_74.11_TM_25.89_ with *e*/*a* = 1.74 and a point density equal to 0.072 Å^−3^. The valance contribution from Al is 3 but the assignment of the TM valence state is complicated. In the present contribution we used the composition-dependent formula by Haüssler (1992 ▸): *e*/*a*_TM_ = 1 − (100 − *x*)/*x*, where *x* is the content of TM. The obtained *e*/*a* value agrees well with complementary values for d-Al–Ni (*e*/*a* = 1.71) and d-Al–Fe (*e*/*a* = 1.78) (Chen *et al.*, 2011 ▸).

The chemical composition of the refined structure model is in very good agreement with the experimental one. Only the point density seems large, almost reaching the upper bound of the Burkov model which is 0.0736 Å^−3^. For instance, the point density of the Kuczera *et al.* model of d-Al–Cu–Co is 0.0659 Å^−3^. The high point density calculated for the model, especially since the calculated Al content exceeds the experimental value, can imply there is no more than one excessive atom, potentially atom 23 from a thin rhombus. Its content grew to almost 80%, even though in the initial electron density map it was much weaker. Unfortunately, the quality of the dataset does not allow us to examine this speculation.

A more detailed analysis of the refinement result is presented in Fig. 7 ▸. The correlation plot of the calculated diffraction amplitudes *F*_calc_ versus the measured amplitudes *F*_obs_ is plotted. The characteristic deviation of the calculated structure factors towards lower values occurring for weak reflections is visible in the form of a ‘tail’. It was already confirmed that this effect is mainly caused by the multiple-scattering which is extremely strong for QCs (Fan *et al.*, 2011 ▸; Buganski *et al.*, 2019 ▸). The error analysis carried out in Fig. 7 ▸ shows the distribution of uncertainties associated with diffraction amplitudes is not uniform. Two sectors for high-intensity peaks and low-intensity peaks can be seen. Unfortunately, We do not know the cause for such behavior as, for example, in the work by Cervellino *et al.* (2002 ▸) a similar plot showed a continuous distribution. The limit of the log_10_(*F*_obs_/σ) normalized to the intensity of the highest peak never reaches below 0, which means that only the peaks with ∣*F*∣ > σ(∣*F*∣) were selected from the dataset. The *R* and *R_w_* values are plotted as a function of the lowest peak intensity in the dataset. For all the peaks *R* = 14.57% and *R_w_* = 5.44%. If the number of weak peaks used for the calculations decreases, the values of the reliability factors also decrease as is typical for QCs.

The basic RPT with an edge length of 2.45 Å is plotted within τ^4^ larger rhombi that were chosen as the basic units of the decagonite. The atoms in both units occupy specific positions within the basic tiling: vertices, mid-edges and positions on the body diagonal dividing the section with the ratio 1:τ. The same properties have already been observed for d-Al–Ni–Rh (Logvinovich *et al.*, 2014 ▸) and d-Al–Ni–Co (Takakura *et al.*, 2001 ▸). Contrary to IQCs, for which the simple-decoration model (Elser & Henley, 1985 ▸) is still useful to discuss the qualitative character of the structure, the atomic decoration of RPT is too random. More useful is a 12.2  Å decagonal cluster which was plotted for each type of rhombus (Fig. 7 ▸). It is located in the position dividing the body diagonal in a τ:1 ratio. The inner pattern of the decagonal cluster was defined as in the Gummelt cluster (Gummelt, 1996 ▸). On the basis of electron microscopy, the inner symmetry of the basic decagonal cluster in d-Al–Ni–Co was established by Saitoh *et al.* (1998 ▸) which is the same as the symmetry of the Gummelt cluster. We decided to test if the same is valid for d-Al–Ni–Fe. The refined atomic decoration shows that the tenfold symmetry of the cluster is broken leaving only the *m* symmetry. By comparing the refined atomic decoration of Gummelt cluster and the one proposed by Steinhardt *et al.* (1998 ▸), we can see that the positions of TM atoms are different. In our case, the TMs are located in the vertices of the brim decagon and in the central kite region. The decoration in the work by Steinhardt *et al.* (1998 ▸) places the TM in the pentagon of the star region and in the centers of the arrows. By this analysis we can conclude that d-Al–Ni–Co is not isostructural to the decagonal structure of decagonite.

## 6. Discussion of the atomic structure   

### 6.1. Projected structure   

**Figure 8:**
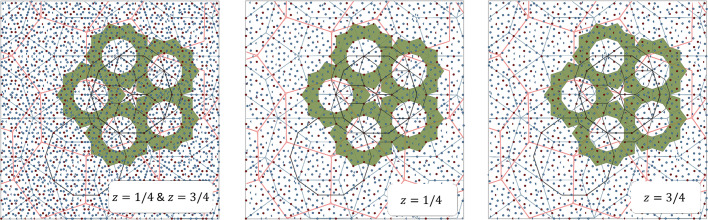
100 × 100 Å sections through the refined structure of decagonite. The RPT and PPT are plotted over the structure for guidance. Two exemplary Hiraga clusters with the subdivision into Deloudi clusters are additionally presented. The formation of pentagonal bipyramids is marked with green pentagons.

The 100 × 100 Å 2D sections, perpendicular to the periodic tenfold direction, through 3D physical-space structure recovered based on refined atomic coordinates, are presented in Fig. 8 ▸. Two PTs are highlighted: RPT (blue), which was originally used to define the building blocks of the structure; and PPT (red) with an edge length of 19.73 Å. Two Hiraga clusters are highlighted. The covering of the structure by Hiraga clusters has already been mentioned, therefore, we did not plot the whole covering. It is worth mentioning that the tenfold symmetry of the Hiraga cluster is broken. Additionally, the orientation of the Hiraga cluster is not unique as both clusters are rotated with respect to each other by 108°. In total, there are ten possible orientations of the Hiraga clusters that constitute the tenfold global symmetry. The decomposition into five 20 Å Deloudi clusters of one Hiraga cluster is also shown.

The characteristic feature of the Al-based DQCs is the formation of pentagonal motives by heavy atoms at each layer and different scales. TM atoms that are linked with a distance of 4.67 Å are highlighted with green pentagons. Together with TM atoms located at every other apex of the Hiraga cluster, TM atoms form pentagonal bipyramids that are important subunits known for periodic approximant crystals of binary DQCs (Steurer, 2004 ▸ *b*). It must be stated that the formation of the pentagonal bipyramids is a consequence of the model and not the initial assumption. For instance, Takakura and coworkers subdivided the atomic surface in a manner that implied the existence of pentagonal motives formed by TM atoms.

### 6.2. Cluster scaling   

In the literature, it is frequently found that a specific cluster was observed in the electron microscopy images. For instance, Saitoh *et al.* (1997 ▸) observed a 20 Å decagonal cluster for d-Al–Ni–Co. Hiraga *et al.* (1991 ▸) proposed a 30 Å cluster for d-Al–Cu–Co, whereas Deloudi *et al.* (2011 ▸) found a 20 Å cluster to be one of the building blocks of the Hiraga cluster. Hiraga *et al.* (1996 ▸) also found a 11 Å decagonal cluster for d-Al–Ni–Fe. Even the cluster with a diameter of 7.6 Å was found for the d-Al–Pd phase (Hiraga *et al.*, 1994 ▸). By carefully studying the diameters of all the mentioned clusters, one can find out that all are related to each other by τ scaling and that in a real structure of a DQC every single one can be found.

**Figure 9:**
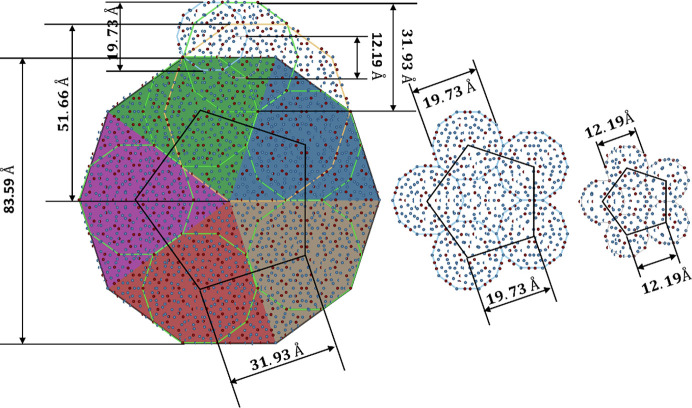
Scaling property of the DQC for decagonite. Decagonal clusters at five different length scales are presented. The characteristic motif of five decagons with a star-like cluster in the center is also shown. This motif is often used to define the atomic decoration of clusters for the cluster-embedding approach.

In Fig. 9 ▸ we show the relations between clusters in the decagonite at different length scales. We start with a large 83.59 Å decagonal cluster that shows a fivefold symmetry. Even though the global symmetry is tenfold, Tsuda *et al.* (1996 ▸) already pointed out that a fivefold symmetry cluster, if properly arranged, can lead to full *P*10_5_/*mmc* symmetry but also different decagonal space groups (*P* 10 *m*2 and *P*10_5_/*m*) observed for d-Al–Ni–Co.

We started with an arbitrary length scale that is related to the 31.93 Å diameter of the Hiraga cluster by τ^2^. It can be seen that a large decagon in each symmetry-dependent sector possesses one Hiraga cluster that outlines a fivefold star in the center. The next in a series of clusters is a 51.66 Å decagonal cluster. This cluster plays for the large decagonal cluster the same role as the Deloudi cluster for the Hiraga cluster. By assembling five such clusters after rotating by 72°, the large 83.59 Å cluster can be restored. After that is a Hiraga cluster, Deloudi cluster and ∼12 Å cluster mentioned by Hiraga *et al.* for the d-Al–Ni–Fe phase.

Furthermore, we present the scaling of the pentagonal motif that is built from five decagonal clusters at three different length scales. We mention this particular motif because two cluster models, with a decagonal cluster and a pentagonal star-like cluster, are often used to establish the initial structure model of the DQC. This was done for the d-Al–Ni–Rh (Logvinovich *et al.*, 2014 ▸) and d-Zn–Mg–Dy (Ors *et al.*, 2014 ▸) structures. In particular the ∼20 Å length scale is often used as it produces a reasonable number of refinable parameters. We show that even for a length scale of ∼12 Å such a motif can be clearly seen. In the case of decagonite the smaller length scale of clusters would be distorted as the decagonal symmetry of the cluster is violated and is statistically driven.

### 6.3. The Gummelt cluster   

**Figure 10:**
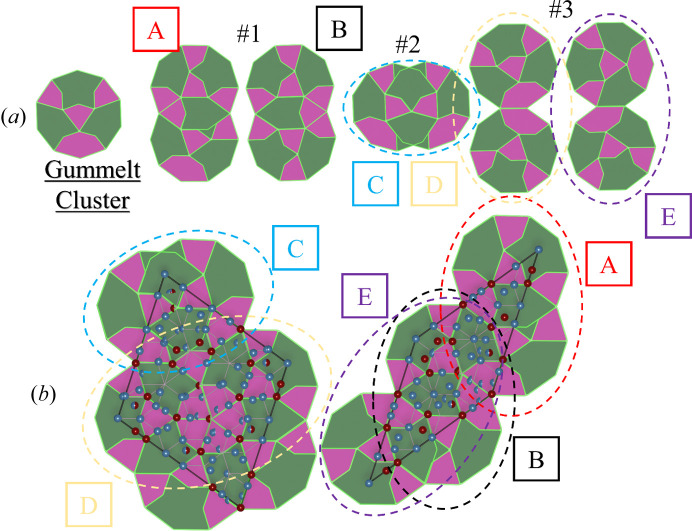
Decoration of the refined units of the RPT with Gummelt clusters. The overlap rules for Gummelt clusters are shown. The *m* symmetry of the decorated Gummelt clusters by atomic species is broken which means that our model cannot be explicitly explained on the basis of Gummelts cluster.

It was speculated by Steinhardt *et al.* (1998 ▸) that the Gummelt cluster [Fig. 10 ▸ (*a*)] can be used as a quasi-unit cell of the DQCs.

In Fig. 10 ▸(*b*) each of the rhombi was decorated with a Gummelt cluster according to the overlap rules. This does lead to three possible ways the clusters can interact within rhombi [Fig. 10 ▸(*a*) #1 to #3]. A thick rhombus intersects seven Gummelt clusters within its volume and a thin rhombus intersects five clusters at that given length scale.

The model of the decagonite structure obtained here cannot be built out of Gummelt clusters explicitly. However, we believe the model could be achieved as the discrepancies are not substantial.

### 6.4. Phononic and phasonic ADPs   

**Figure 11:**
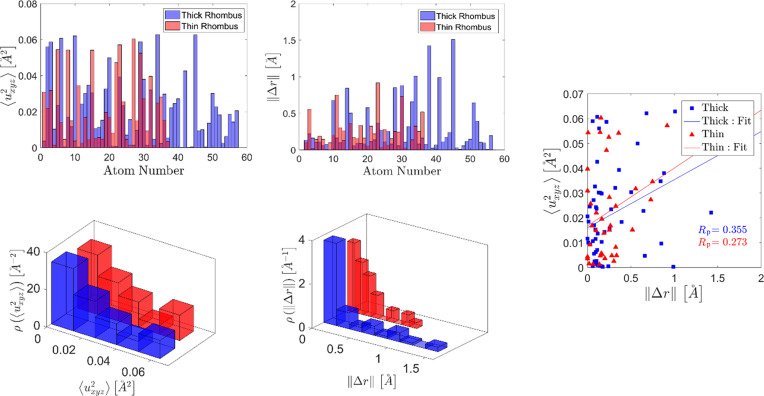
Mean displacement parameter and atomic shift after the refinement for each atom in the asymmetric part of the model. The distribution of the parameters is plotted below with red assigned to the thin rhombus and blue for the thick rhombus. The correlation between the magnitude of the atomic shift 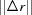 and the phononic mean displacement parmeter 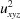 is presented. For both units a weak positive correlation is found.

The refined structure model of decagonite shows a rather small chemical and positional disorder, comparable to known models of the d-phase. The maximal atomic mean square displacement parameter 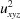, related with *B* factors according to the formula 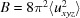 is equal to 0.062 Å^2^. Such a value is not exceptional for QCs and even larger mean displacements were reported, reaching 0.076 Å^2^ in d-Al–Ni–Co (Kuczera *et al.*, 2012 ▸). In Fig. 11 ▸ the atomic mean square displacement is plotted for each atom from the asymmetric parts of thick and thin rhombi. The atoms are numbered according to Table S1 of the supporting information. Also, the distribution of mean displacement is plotted with a bin size optimized according to the Freedman–Diaconis rule (Freedman & Diaconis, 1981 ▸). We attempted to investigate the shape of the distribution for atomic ADPs but the statistics are too small. It is known that for macromolecules the distribution has the shape of the shifted inverse gamma distribution (Masmaliyeva & Murshudov, 2019 ▸). The only conclusion we can make is that the majority of mean atomic displacements are below 0.02 Å^2^. For both types of rhombi the increase in the number of atoms manifesting 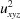 above 0.05 Å^2^ is noted.

The correlation between the shift of the atom from the initial position of the refinement and the mean displacement parameter is now discussed. From Fig. 11 ▸ it can be seen that the maximal displacement is estimated slightly above 1.5 Å in the thick rhombus. The distribution of the atomic shifts clearly shows that many atoms do not move beyond 0.2 Å. There is a small positive correlation between atomic shift and mean phononic displacement. The correlation is not strong, with a Pearson correlation coefficient *R*_P_ = 0.355 for a thick rhombus and *R*_P_ = 0.27 for a thin rhombus.

**Figure 12:**
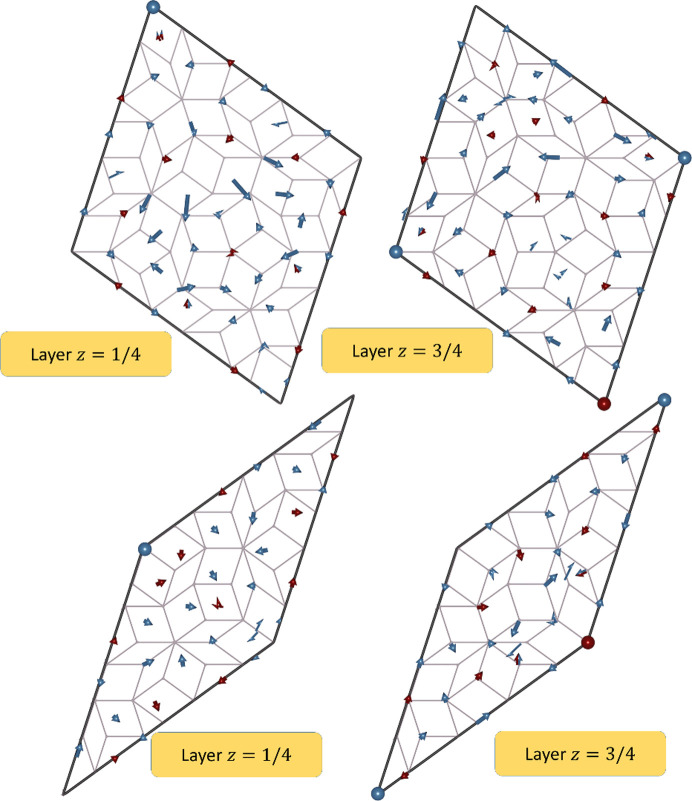
Vector and magnitude of the atomic shift after the refinement from the initial position. The colors of the arrows refer to the atomic species (following the same scheme as in Fig. 2 ▸).

The direction of movement is plotted in Fig. 12 ▸: arrows point from the initial position toward the refined one. The magnitude of the displacement is indicated by the size of the arrow. Because some arrows would have to be smaller than the tip of an arrow we decided to indicate the magnitude by the length of an arrow without the tip. If an atom did not move, we placed in this location a ball with a color indicating the type of atomic species (color coding following that in Fig. 6 ▸).

The phason atomic displacement was accounted for in the form of the general Debye–Waller formula 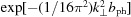, where *b*_ph_ is the phasonic *B* factor. For the refined structure of decagonite it was estimated to be 3.88 Å^2^. However, is it best to present the *b*_ph_ coefficient in unit-free form. In our case the value is 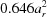. It is a rather low value taking into account the fact that the structure grew naturally. Synthetic d-Al–Cu–Co shows 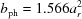 – more than two times higher. The result is comparable to the best known structure model of the DQC which is d-Al–Cu–Rh with 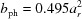. On this basis we could conclude that the magnitude of random phason disorder in the sample of decagonite is not beyond observed values for synthetic QCs.

### 6.5. Higher-dimensional representation   

**Figure 13:**
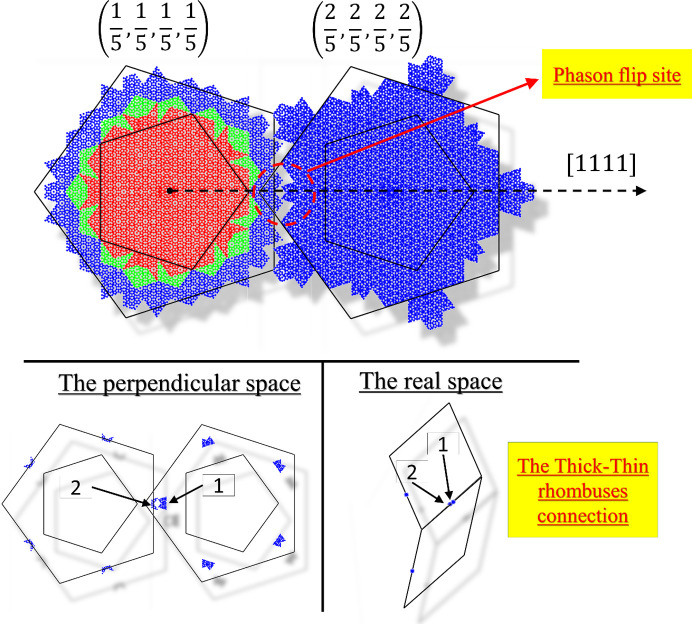
Recovered atomic surfaces for the decagonite structure. Red –TM atoms, blue – Al atoms and green – mixed atoms. The partially occupied sites are not differentiated in this image. The phason flip domain in the atomic surfaces is highlighted by the red circle. The domain corresponds to Al atoms located at the edges of rhombi.

In order to lift the structure to 4D a large portion (>500 000) of atomic positions was generated. All positions were represented as 4D vectors. After multiplying the 4D vector of each atom by the inverse projection matrix **W** ^−1^, the coordinates in 4D space were found. The coordinates were then reduced to one 4D unit cell by the modulo 1 operation. Every position of the generated structure was then assigned to the corresponding atomic surface. The assignment is, however, not deterministic. In this work, the atom is allocated to the closest atomic surface with the distance calculated in 4D space. The recreated atomic surfaces are plotted in Fig. 13 ▸ to allow for comparison with pentagons of the RPT. The fractional sites are not marked differently. All TM atoms are located in the first pentagon which fully agrees with the *ab initio* structure solution by *SUPERFLIP*. No atoms are assigned to the atomic surface in the origin of the 4D unit cell which can be attributed to empty positions corresponding to centers of Hiraga clusters.

The atomic surfaces centered at (1, 1, 1, 1)/5 and (2, 2, 2, 2)/5 should ideally match on the brink of the distributions. In our case there is a small discrepancy marked with a red circle in Fig. 13 ▸. The regions overlap, therefore the closeness condition (Frenkel *et al.*, 1986 ▸) is violated. The overlap indicates that two closely laying atoms have been created: this is a phason flip site. Only the connection between thick and thin rhombi is problematic as two adjacent rhombi of the same kind do not create a split atom. These two atoms are counted in the structure factor calculations with a fraction 0.5, meaning even though the atomic surfaces overlap, they add up to one full atom.

**Figure 14:**
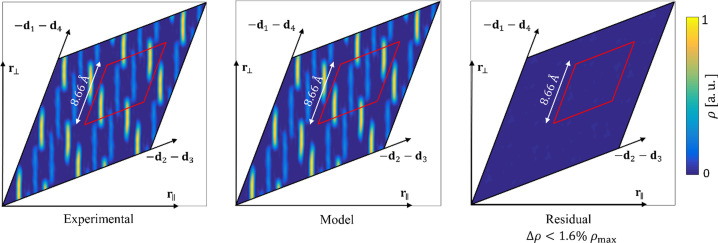
2D section through the electron density map recovered on the phases of the peaks coming from the refinement. The experimental amplitudes, coming from the model and their differences, were used to recover density maps. The residual electron density is lower than the potential atom, therefore we can conclude that our model does not miss any atom.

The last test of our model was the comparison between the calculated high-symmetry sections through 4D space. The phases obtained from the refined structure serve to calculate the electron density map using experimental diffraction amplitudes and those recovered from the model (Fig. 14 ▸). By calculating the residual electron density ∣Δρ∣ < 1.6%ρ_max_ we can be sure that no atoms are missing in the structure.

## 7. Conclusions   

Quasicrystals are usually synthesized in the laboratory by mixing precise ratios of selected elemental components in liquid and quenching under strictly controlled conditions ranging from rapid to moderately slow. Nonetheless, the finding of two natural quasicrystals in the Khatyrka meteorite which displays clear evidence of a shock generated by a high-velocity impact event introduced a dramatic new possibility as to how these materials might be formed. Here we have obtained the first structural model for a natural quasicrystal and showed that it does not exhibit structural peculiarities that would significantly differ from synthetic quasicrystals. The structure very much resembles the structural model known for Al–Cu–*M*, (*M* = Co, Ir, Rh). Due to the quality of the diffraction data, we could not unambiguously determine whether the linear phason strain is present or absent in the sample. The HRTEM image of the selected region of the sample does not indicate the linear phason strain to be present. Surprisingly, based on the refinement, the random phason disorder is comparable to the best synthetic quasicrystals. Even more, the phasonic *b*_ph_ is comparable in value to the best structural model known for d-Al–Cu–Rh. Also, phonon ADPs are standard in magnitude. It is interesting to note that no puckering of atoms from atomic layers is observed for synthetic QCs. Since this is the only example of a structural model of a natural DQC such a feature cannot yet be generalized.

The crystallographic *R* factor we obtained for decagonite is 14.57% for peaks with amplitudes ∣*F*∣ > σ(*F*). It is comparable to that obtained for the synthetic Al–Ni–Co decagonal phase. Lower values of *R* have been reported for decagonal phases in the literature but they require the use of reflections with ∣*F*∣ > 3σ(*F*), which are generally more reliable. The excellent value of the weighted *R_w_* = 5.44% for decagonite indicates that the high value of *R* is caused by a high-θ data error rather than potential flaws in the structural model.

## Supplementary Material

Crystal structure: contains datablock(s) I. DOI: 10.1107/S2052252520015444/yu5021sup1.cif

Structure factors: contains datablock(s) Decagonite. DOI: 10.1107/S2052252520015444/yu5021sup2.hkl

Table S1: list of the structure parameters after the refinement. DOI: 10.1107/S2052252520015444/yu5021sup3.pdf

CCDC reference: 2045893
