# Supplementary material for: Insight into the structure of decagonite – the extraterrestrial decagonal quasicrystal
Source: IUCrJ. 2021 Jan 1;8(Pt 1):87–101. doi: 10.1107/S2052252520015444 (PMC7792992; doi:10.1107/S2052252520015444)
Supplement: Supplementary file 3 [file m-08-00087-sup3.pdf]

IUCrJ

Volume 8 (2021)

Supporting information for article:

Insight into the structure of decagonite – the extraterrestrial decagonal quasicrystal

Ireneusz Buganski and Luca Bindi

Table S1 contains the list of the refined parameters. Coordinates x, y and z are given in a rhombohedron-spanning base, which means that the real-structure **r** coordinate vector is obtained as follows: $r = X\mathbf{f}_1 + Y\mathbf{f}_2 + Z\mathbf{f}_3$, where $\mathbf{f}_1, \mathbf{f}_2, \mathbf{f}_3$ are vectors spanning edges of the rhombohedra. For the AR those vectors are $\mathbf{a}_1$, $\mathbf{a}_2$, $\mathbf{a}_6$ respectively and for the OR $\mathbf{a}_1$, $-\mathbf{a}_3$, $-\mathbf{a}_6$. The letter 'L' stands for an acute rhomb and the letter 'S' stands for a obtuse rhombus. SOF defines site occupancy factors: 1 means the position is fully occupied, 0 means it is empty (vacant site). The column labelled "Fraction" gives the ratio of atom inside a rhombus depending on it position in a rhombus f. i. atom on the edge is only in half inside a rhombus. Variable $\langle u^2{}_{xyz} \rangle$ defines the mean atomic displacement parameter. The variables p_{Al} and p_{TM} define probabilities that the atomic sit eis occupied by Al or TM respectively.

Table S1. The list of the structure parameters after the refinement.

Rhombi type	Atom number	x	y	z	p_{Al}	p_{TM}	SOF	$\langle u^2{}_{xyz} \rangle$	Fraction
L	1	0	0	3.07875	0	1	1	0.003774	0.10
L	2	0.08194	0	1.02625	1	0	0.958392	0.056037	0.50
L	3	0.151281	0	3.07875	1	0	1	0.058678	0.50
L	4	0.228056	0	1.02625	0	1	1	0.010315	0.50
L	5	0.468462	0	3.07875	1	0	1	0.023639	0.50
L	6	0.608463	0	1.02625	1	0	1	0.060683	0.50
L	7	0.76882	0	1.02625	0	1	1	0.000611	0.50
L	8	0.855836	0	3.07875	0	1	1	0.017027	0.50
L	9	1	0	3.07875	1	0	1	0.01162	0.30
L	10	0.278428	0.08664	3.07875	1	0	1	0.062131	1.00
L	11	0.481335	0.098764	1.02625	0.986326	0.013674	1	0.000517	1.00
L	12	0.710557	0.090267	3.07875	1	0	1	0.024278	1.00
L	13	0.850315	0.089097	1.02625	1	0	1	0.030174	1.00
L	14	0.502517	0.160469	1.02625	1	0	0.237625	0.034789	1.00
L	15	1	0.152381	3.07875	1	0	0.00463	0.030331	0.50
L	16	0.148334	0.14482	1.02625	1	0	1	0.010562	0.50
L	17	0.28818	0.153222	1.02625	1	0	1	0.011441	1.00
L	18	0.381717	0.152733	3.07875	0	1	1	0.015251	1.00
L	19	0.525972	0.143533	3.07875	1	0	1	0.032605	1.00
L	20	0.652667	0.150623	1.02625	1	0	1	0.049962	1.00
L	21	0.916319	0.141718	3.07875	0.68301	0.31699	1	0.000909	1.00

L	22	1	0.155653	1.02625	1	0	1	0.000696	0.50
L	23	0.251133	0.250414	3.07875	1	0	1	0.039242	0.50
L	24	0.363668	0.242461	1.02625	1	0	1	0.023391	1.00
L	25	0.613096	0.245042	3.07875	0	1	1	0.007068	1.00
L	26	0.775279	0.225206	3.07875	1	0	1	0.015616	1.00
L	27	0.850775	0.243192	1.02625	0	1	1	0.001379	1.00
L	28	0.701627	0.237742	1.02625	1	0	1	0.009603	1.00
L	29	0.524875	0.297975	1.02625	1	0	1	0.058912	1.00
L	30	0.919324	0.246969	3.07875	1	0	1	0.038002	1.00
L	31	0.327841	0.316493	3.07875	1	0	0.490549	0.001355	0.50
L	32	0.463754	0.347678	3.07875	1	0	0.137661	0.032026	1.00
L	33	0.917268	0.361999	1.02625	1	0	0.366558	0.004648	1.00
L	34	0.777369	0.319314	1.02625	1	0	1	0.062845	1.00
L	35	0.379068	0.382033	1.02625	0	1	1	0.002451	0.50
L	36	0.622635	0.381084	3.07875	1	0	1	0.029746	1.00
L	37	1	0.382442	3.07875	0	1	1	0.005507	0.50
L	38	0.604016	0.397359	1.02625	1	0	1	0.022106	1.00
L	39	1	0.467295	1.02625	1	0	1	0.02448	0.50
L	40	0.47109	0.475844	1.02625	1	0	1	0.027155	0.50
L	41	0.673794	0.530538	3.07875	1	0	1	0.000242	1.00
L	42	0.853021	0.483473	3.07875	1	0	1	0.042587	1.00
L	43	0.756431	0.527213	1.02625	0	1	1	0.000208	1.00
L	44	0.520661	0.52327	3.07875	0	1	1	0.000419	0.50
L	45	1	0.614407	3.07875	1	0	1	0.062636	0.50
L	46	0.856636	0.611781	1.02625	1	0	1	0.009264	1.00
L	47	1	0.613917	1.02625	0	1	1	0.010042	0.50
L	48	0.759132	0.622506	3.07875	0	1	1	0.000531	1.00
L	49	0.836988	0.642734	3.07875	1	0	0.403811	0.01362	1.00
L	50	0.917726	0.674583	3.07875	1	0	1	0.030071	1.00
L	51	0.688447	0.683303	1.02625	1	0	1	0.022739	0.50
L	52	0.748158	0.776204	3.07875	1	0	1	0.018299	0.50
L	53	0.904027	0.766059	1.02625	1	0	1	0.002575	1.00
L	54	0.853301	0.852635	3.07875	0	1	1	0.006215	0.50

L	55	1	0.851034	3.07875	1	0	1	0.018486	0.50
L	56	0.924249	0.913515	1.02625	0.63107	0.36893	1	0.01429	0.50
L	57	1	1	1.02625	1	0	1	0.020543	0.10
S	1	0	1	3.07875	1	0	1	0.030858	0.10
S	2	0	0.858997	3.07875	0	1	1	0.021999	0.50
S	3	0.1864	1	1.02625	1	0	1	0.031729	0.50
S	4	0	0.766623	1.02625	0	1	1	0.001518	0.50
S	5	0.139288	0.843343	3.07875	1	0	1	0.054454	1.00
S	6	0.385168	1	3.07875	0	1	1	0.01382	0.50
S	7	0.22214	0.844559	1.02625	1	0	1	0.004681	1.00
S	8	0	0.611903	1.02625	1	0	1	0.054285	0.50
S	9	0.467613	1	1.02625	1	0	1	0.014832	0.50
S	10	0.245106	0.768282	3.07875	1	0	1	0.003648	1.00
S	11	0	0.486318	3.07875	1	0	1	0.034535	0.50
S	12	0.534802	1	3.07875	1	0	1	0.002564	0.50
S	13	0.128798	0.610775	1.02625	0	1	1	0.016826	1.00
S	14	0.616325	1	1.02625	0	1	1	0.004258	0.50
S	15	0.38276	0.748347	1.02625	1	0	1	0.054148	1.00
S	16	0.236894	0.61048	3.07875	0	1	1	0.00521	1.00
S	17	0.508307	0.838945	3.07875	1	0	1	0.005669	1.00
S	18	0.252419	0.531458	1.02625	1	0	1	0.004811	1.00
S	19	0.607633	0.840947	1.02625	1	0	1	0.019524	1.00
S	20	0.378932	0.616083	3.07875	1	0	1	0.016772	1.00
S	21	0.171874	0.392117	3.07875	0.793354	0.206646	1	0.008177	1.00
S	22	0	0.238953	1.02625	0	1	1	0.047206	0.50
S	23	0.246835	0.45351	3.07875	1	0	0.816895	0.057127	1.00
S	24	0.855827	1	3.07875	1	0	1	0.025689	0.50
S	25	0.600432	0.734822	3.07875	0	1	1	0.004775	1.00
S	26	0.234501	0.377153	1.02625	1	0	1	0.001279	1.00
S	27	0	0.155245	3.07875	1	0	1	0.060296	0.50
S	28	0.758966	0.834845	1.02625	0	1	1	0.015054	1.00
S	29	0.52177	0.603082	1.02625	1	0	1	0.05252	1.00
S	30	0.348025	0.476169	3.07875	1	0	1	0.027291	1.00

S	31	0	0.079555	1.02625	1	0	0.962965	0.009206	0.50
S	32	1	1	1.02625	1	0	1	0.039679	0.20
S	33	0.747507	0.741758	3.07875	1	0	1	0.001206	0.50
S	34	0.411471	0.416751	1.02625	0	1	1	0.024708	0.50
S	35	0.244042	0.251055	3.07875	1	0	1	0.028208	0.50
S	36	0.118789	0.144911	1.02625	1	0	0.994502	0.015157	0.50
S	37	0	0	3.07875	0	1	1	0.003483	0.20
